# Old age and other factors associated with salivary microbiome variation

**DOI:** 10.1186/s12903-021-01828-1

**Published:** 2021-10-04

**Authors:** Joel L. Schwartz, Natalia Peña, Nadia Kawar, Andrew Zhang, Nicholas Callahan, Steven J. Robles, Andrew Griebel, Guy R. Adami

**Affiliations:** 1 Department of Oral Medicine and Diagnostics, College of Dentistry, University of Illinois at Chicago, 801 South Paulina Street, Chicago, IL 60612 USA; 2 Department of Periodontics, College of Dentistry, University of Illinois at Chicago, Chicago, IL USA; 3 Department of Oral and Maxillofacial Surgery, College of Dentistry, University of Illinois at Chicago, Chicago, IL USA

**Keywords:** Aging, Polypharmacy, Saliva microbiome, Periodontal disease

## Abstract

**Background:**

Many factors can contribute to the exact makeup of the salivary microbiome. Differences in the oral microbiome occur with old age, which may be due to oral conditions and diseases associated with old age, such as edentulism, as well as other unknown causes.

**Methods:**

The salivary microbiome was sampled in patients from a large urban clinic. For all subjects age, gender, periodontal status, caries status, presence of edentulism, medications, and tobacco usage were recorded. Multifactor analysis was used to study variation in salivary microbiome profiles linked to these factors.

**Results:**

In the population sampled, there were significantly higher numbers of edentulous subjects, and increased levels of polypharmacy found with aging. Large differences in alpha diversity and beta diversity of the salivary microbiome in the old age group were largely linked to edentulism. However, multivariable analysis revealed, even after adjusting for differences in edentulism, polypharmacy, tobacco usage, periodontal disease, caries level, and gender, that old age itself was associated with lower levels of taxa *Porphyromonas endodontalis, Alloprevotella tannerae*, *Filifactor alocis*, *Treponema*, *Lautropia Mirabilis* and Ps*eudopropionibacterium sp._HMT_194.* Surprisingly, of these taxa, most were ones known to reside on or near tooth surfaces.

**Conclusions:**

Another factor or factors beyond edentulism, polypharmacy and periodontal disease play a role in the differences seen in oral microbiome with old age. The nature of this factor(s) is not known.

**Supplementary Information:**

The online version contains supplementary material available at 10.1186/s12903-021-01828-1.

## Background

“Aging is the progressive loss of physical integrity that culminates in reduced function” [1]. Changes to cellular macromolecules can alter cell function, changing histology and physiology of tissue, and resulting in changes in organ function. Senescence is the end stage of this process, a state where tissue function is so altered or degraded that maintenance of homeostasis is limited.

In old age there can be many changes in oral tissue. The mucosa can decrease in cellularity and show changes in extracellular matrix [2]. Salivary glands can change, resulting in reduced production and secretion of saliva [3, 4]. Changes in the systemic immune system can result in immune cells with impaired migratory ability, resulting in changes in innate immunity response to perturbation in the oral cavity. “Immunosenescence” is the loss of naive and regulatory B and T cells that occurs with old age, which can lead to altered immunity and poorer resolution of inflammation [5]. Clinically observed increase in periodontal disease and loss of teeth are common occurrences in old age [6, 7]. While these effects can alter cell, tissue, and organ function, they also can contribute to change in the co-existing oral microbiome which in turn can contribute to the state of health and disease in the mouth, throat, and elsewhere in the body. There are examples of gut bacteria that encode enzymes that synthesize short chain fatty acids, or convert tryptophan to aryl hydrocarbon receptor ligands, which can alter inflammation and/or mucosal integrity [8, 9]. Similarly, bacteria in the oral cavity may contribute to oral immune and inflammation state, making understanding how bacteria changes with age crucial to understanding oral tissue function in old age.

There is evidence that the overall oral microbiome, as measured in saliva and oral surface, changes with old age. In some studies, measures were taken to separate these changes from differences in oral health that occur with aging such as periodontal disease prevalence [6, 10]. Among high throughput studies working with subjects older than 60 or 70 years of age, Xu et al. examined saliva from subjects controlled for periodontal disease and full dentition, and showed older subjects, 62–76 years of age, had lower levels of *Haemophilus* than young adult subjects, among other differences [11]. This small study contained about 11 subjects per group. In a study that examined general health of subjects of 19–33, 68–88 and > 100 years of age, Wu et al. saw a reduction in species richness in centenarians but found few other changes, though the lower level of *Haemophilus* was again noted in subjects over 68 years old [12]. This study did not measure differences in oral health and was limited in sample size to about 20 subjects per group. In a large study, Takeshita et al. defined two salivary microbial population types. One rich in *Prevotella*, *Veillonella, Actinomyces, Rothia. S. Salivarius* and *S. Parsanguinas*, favored older adults, while a second rich in *Neiseria, Hameophilus, Porphyromonas, M. Gemelli and S. Mitis* was prominent in the young after correction for body mass index, caries, tobaccos use, number of teeth, periodontal disease and plaque levels [13]. Generalizing the data is made difficult by the different approaches used to deal with variation in oral health and additional subject characteristics, and variation due to study location [14]. Importantly, this last study concluded that there were changes in salivary microbiome that were not due to intraoral disease [13].

While recent and much earlier studies point to differences in the salivary and oral surface microbiome of old subjects most studies have been small and difficult to generalize. In this study, we used next generation sequencing methods to characterize taxa in the saliva of a population at an urban dental clinic in the United States. This was combined with high quality data on clinical covariates to attempt to isolate effects of age after correction for effects of edentulism, periodontal disease, and polypharmacy, factors well known to increase with old age. The inclusion criteria included age greater than 18 years, near full dentition (20 or more teeth), or edentulism, lack of dental implants. The null hypothesis is that there will be no taxa differences between old and young saliva microbiome that withstand correction of the tested clinical covariates of the aged such as increased edentulism.

## Methods

### Study population and patient characteristics

A cross-sectional study was conducted at the University of Illinois at Chicago College of Dentistry, General Practice and Denture clinics between 11/08/2016 and 06/21/2019 [15]. All subjects provided written informed consent to participate in accordance with guidelines of the institutional ethics committee of the University of Illinois at Chicago, Institutional Review Board 1, which approved this study #2016-0696. This study was done in full accordance of the principles of the Declaration of Helsinki. Study inclusion criteria were: 18 years of age and older, medical record, current medication list, full periodontal exam, visual, tactile and radiographic caries exam, dentate or edentulous, and agreement to supply a saliva sample. Study exclusion criteria were: presence of restored dental implants, removable partial dentures, maxillofacial defects, scaling of teeth within the past 3 months; acute disease that requires urgent care, less than twenty [20] natural teeth for the dentate subjects, antibiotic use within the past month. Zaura et al. [16], use of antimicrobial mouthwash within the past 48 h, and food consumption within the past 1 h. Periodontal health was assessed using the ADA/AAP classification system with Classes I and II considered healthy or having mild form of periodontal disease. Class III having moderate and Class IV having severe periodontal disease. Edentulous patients without periodontia were considered to lack active periodontal disease. The criteria for dental caries were applied using World Health Organization recommendations, which describe caries as lesions of the tooth’s surface with an unmistakable cavity, undermined enamel, or a detectably softened floor or wall [17]. The levels of caries represents the number of tooth surfaces involved. A subset of samples were reported on in an earlier study [18].

### Sample collection

Stimulated saliva was collected from patients asked to chew paraffin over a 5-min period as described [19]. Saliva was kept on ice for less than 2 h prior to centrifugation at 8000×*g*, washing the pellet 2× with cold PBS, then storage of the pellets at − 80 °C.

### Quantitation of microbial abundance

Quantitative PCR analysis was performed to determine the relative abundance of microbial 16 S rRNA genes in 60 saliva sample extracts. Amplification reactions were performed as described previously [20] using a CFX Connect Real Time PCR System (Bio-Rad).

### Characterization of microbial community structure

Genomic DNA was extracted from saliva samples using ZR Fungal/ Bacterial DNA MiniPrep D6005 (Zymo Research Corp, Irvine, CA, USA) with the Biopsec Mini Beadbeater for homogenization (BioSpec Products Inc, Bartlesville, OK, USA) using two separate treatments for 80 s, separated by cooling on ice. The amplicon assay targeted the V1–V3 variable region of bacterial 16S ribosomal RNA rRNA genes using the primer sets 27F/534R [19]. This was followed at the University of Illinois at Chicago Sequencing Core by a second PCR amplifcation when sample specific barcodes were added as described previously [21]. In preparation of sequencing the samples unincorporated primers were purified away using SequalPrep Normalization Plate Kit (Applied Biosystems) followed by Qubit (Invitrogen) based sample quantification. Equal amounts of each sample are pooled. To increase sample complexity which is a requirement of DNA sequencing of amplicon samples on an Ilumina sequencer, a 20% spike in of PhiX viral DNA library is added. Negative controls were samples that started with H2O instead of saliva DNA. Additional controls were technical replicates from several donors.

For taxa assignment and measurement, reverse sequences from the FASTQ files were analyzed using the software package QIIME2 [22–24]. Sequences were trimmed if the average quality was lower than 25. As a result, the read sequences were truncated at 252nt. DADA2-plugin in QIIME2 was used to sequence, denoise, and generate feature data and feature tables for the dataset [25]. It has earlier been shown that sequencing of V3 of 16S rDNA can be used to differentiate oral taxa when aligned to the HOMD annotated sequences [26]. Taxonomy assignment was done by classify-consensus-blast function with 98% match identity to the Human Oral Microbiome Database [27]. Of the 271 samples, there were 13,383–63,154 reads per sample. There were 10.4 million reads total. Data from 5 additional subject samples were discarded due to read numbers at less than 9000 or read profiles resembling DNA free negative controls [28].

### Statistical analysis

Alpha diversity analysis was performed using MicrobiomeAnalyst [29]. This analysis included calculation of Shannon’s diversity index of both species number and their distribution, and Chao1 indices of richness. Beta diversity analysis was visualized using Bray–Curtis dissimilarity (non-phylogenetic) metric. ADONIS, or permutational multivariate analysis of variance using distance matrices, [30] was used to find association between clinical metadata and beta diversity of taxa abundance in the samples as accessed through QIIME2 [22]. ANOVA was performed using Kaleidagraph 4.1.2 (Synergy Software, Reading, PA USA).

MaAsLin2 is a tool that allows the determination of multivariable associations between clinical metadata and microbiome data using a boosted, additive general linear model [31]. Covariates included, gender, age, whether patient was dentate or edentulous, tobacco use, caries count, and periodontal status. All taxa considered were non-zero in 25% of samples.

## Results

### Study population

A total of 271 patients with medical records and periodontal examinations at the University of Illinois at Chicago College of Dentistry Clinics were included in this study. The population was divided by age, with mean age of 33.72 years in the young group (N = 85), 56.59 years in the middle age group (N = 101), and 72.98 years in the old age group (N = 85). Both the old and middle age groups significantly differed in dentate status compared to the young group, with 19.8% of the middle age group and 45.9% of the old group being edentulous. Medication use was also associated with increasing age as the average medication use was 1.3 ± 2.53 medications in the young group, 3.46 ± 4.42 medications in the middle age group, and 5.08 ± 6.46 in the old group. The old age group was shown to have high levels of both edentulism and polypharmacy, or the consumption of at least five medications at the same time (Table 1). Compared to the young group, significant differences in active periodontal disease were revealed only in the middle age group. The incidence of current periodontal disease in the old group was attenuated by the high number of subjects with no teeth. The old group had fewer active caries for the same reason. Finally, there were no significant differences in tobacco use in the middle age and old groups compared to the young group though there was a trend toward a difference in gender.

**Table 1:** Demographics of the study population

Demographics		Young (18–45)	Middle age (46–64)	*p* value	Old (65–94)	*p* value
		N = 85	N = 101		N = 85	
Gender*	Female	59	56		49	
	Male	26	45	*p* < 0.0688	36	*p* < 0.1513
Tobacco use*	Yes	11	21		17	
	No	74	80	*p* < 0.1765	68	*p* < 0.3012
Periodontal disease*	Yes	21	43		23	
	No	64	58	*p* < 0.0131	62	*p* < 0.8611
Dentate*	Yes	82	81		46	
	No	3	20	*p* < 0.0006	39	*p* < 0.00001
Caries** ***	Mean	4.87 ± 0.85	3.82 ± 0.73	*p* < 0.35	1.3 ± 0.35	*p* < 0.0001
Age**	Mean	33.7 ± 1.3	56.6 ± 5.1	*p* < 7.47 × 10^−54^	73.0 ± 6.5	*p* < 1.006 × 10^−82^
Medication Count**	Average	1.31 ± 2.53	3.46 ± 4.42	*p* < 2.55 × 10^−5^	5.08 ± 6.46	*p* < 2.36 × 10^−11^

*Fisher exact test versus young

**Student *t*-test versus young

***2 subjects in Young and 1 in Middle age group unknown caries level

### Quantitation of levels of total bacterial marker DNA per sample

An interesting question is what are the relative levels of bacteria in the different subject groups. An examination of salivary bacterial DNA levels in a subset of dentate subjects from each age group, young, 18–45, middle age, 46–64, and old age, 65–94, was done using qPCR. This revealed relative levels of 0.43 ± 0.18, young, 0.080 ± 0.025 middle age and 0.113 ± 0.045 old age groups. A oneway ANOVA was done to compare levels of total bacteria 16S rRNA DNA in the three groups. There was no significant difference for the 3 groups: F(2, 58) = 0.785, *p* < 0.46.

### Diversity of the salivary microbiome

**Fig. 1:**
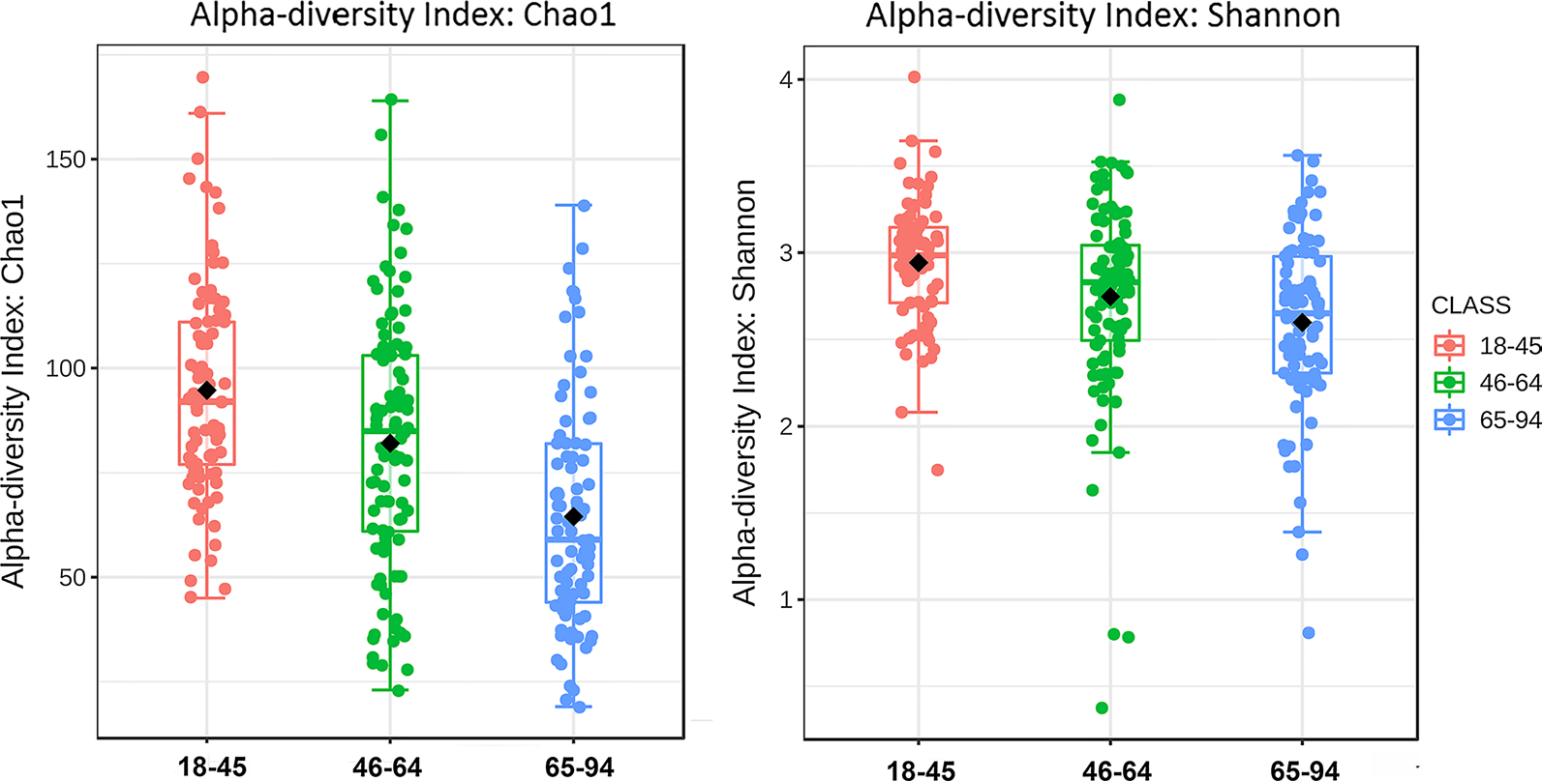
Phylogenetic Diversity of saliva from young, middle, and old aged groups. Box plot of: **a** comparison of richness based on the Chao1 indices shows middle and old age groups show lower diversity than the young group, *p* < 9.69 × 10^−13^ and *p* < 6.52 × 10^−05^, respectively; **b** comparison of Shannon Diversity reveals the old age group is less diverse than young group *p* < 1.5 × 10^−05^ and trends that way for middle age versus young, *p* < 0.0701

Alpha diversity, in the form of the Chao1 index, is a measure of the level of variation of taxa within a sample, based on taxa richness. The Shannon Diversity index is another measure of alpha diversity. It combines taxa richness along with the evenness of the spread of taxa. Both Chao1 and Shannon index measures revealed statistically significant differences in intra-sample diversity between age groups, with a decrease in diversity with increased age (Fig. 1). Chao1 measurements showed species richness was significantly lower in the old age group compared to the young group (*p* = 9.69 × 10^−13^) and middle age group (*p* = 6.52 × 10^−05^). This parallels Shannon measurements of species richness and evenness with significance values of *p* = 1.50 × 10^−06^ in the young versus old age groups, and trending toward significance with the young versus middle age groups at *p* = 0.0701.

**Fig. 2:**
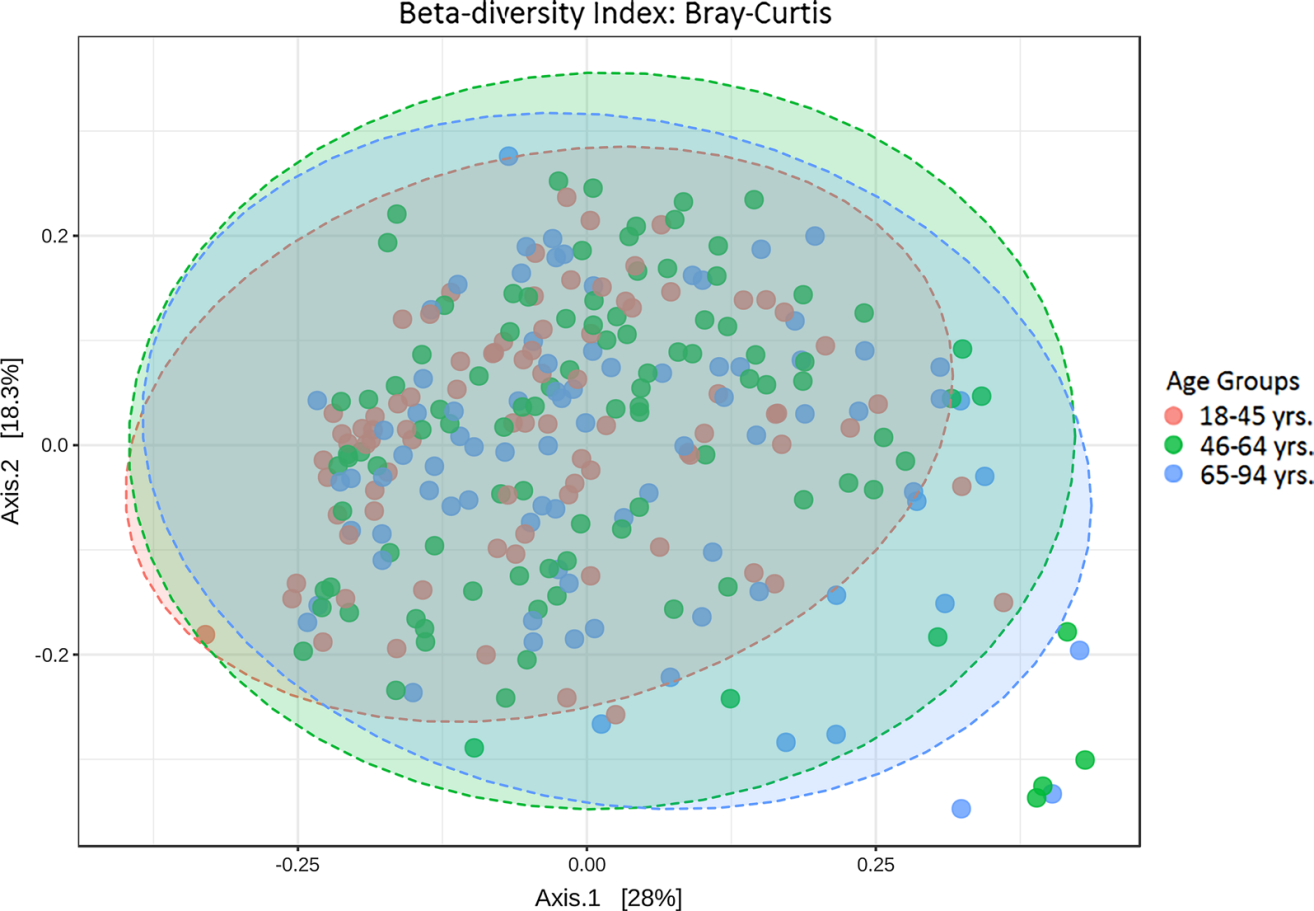
Principal coordinate analysis (PCoA) of saliva microbiome profiles of all 3 groups based on Bray–Curtis distances

Further analysis of microbial diversity uncovered differences between the age groups by principal coordinate analysis plot with slight cluster separation of age groups in Fig. 2. Beta diversity is a measure of variation of specific species between samples. The Bray–Curtis distance of the composition of taxa revealed statistically significant differences in composition between the young and old age groups based on PERMANOVA: R^2^ = 0.0223, and *p* < 0.002. The same test revealed a difference between the young and middle age groups, R^2^ = 0.0126 and *p* < 0.022 but not between the middle and old age groups, R^2^ = 0.00661 and *p* < 0.237. Additional file 1: Fig. S1 provides insight to the specific taxa that are found at different levels in the old age group and the counger groups.

**Fig. 3:**
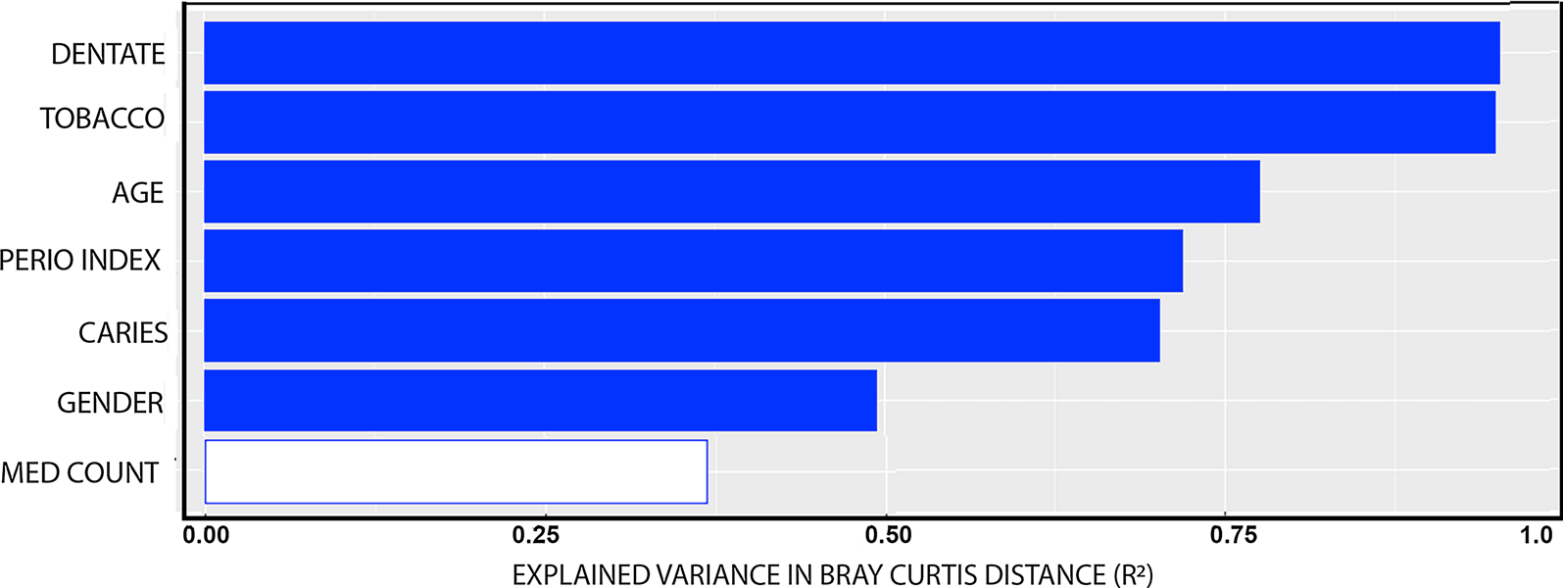
Schematic of variables that are associated with variation of Bray–Curtis distances of the 3 groups. The x-axis represents the percentage of variance in the Bray–Curtis distance that are explained by the 6 variables. Blue bars are for factors that significantly explain variation in salivary taxa (*p* < 0.05). No value surpassed 1%

A permutation multivariate analysis of variance using distance matrices (ADONIS) was done to assess the contribution of each clinical variable to the overall variation seen in the Bray–Curtis distances between different samples in the whole population. This analysis revealed that of the 7 clinical covariates, 6 contributed to the variation in taxa among the saliva samples though at fairly low levels (Fig. 3). The fact that a patient was dentate versus edentulous and whether they used tobacco contributed most significantly to the variation in taxa observed within the whole population, about 1% each (*p* < 0.001 for both). Age and active caries level contributed at a lesser significance to variation in taxa, followed by presence of poor periodontal status, and a small contribution from gender (*p* < 0.001, *p* < 0.002, *p* < 0.002, and *p* < 0.023, respectively). Medication count failed to show a significant role in variation by this measure (*p* < 0.331).

### Composition of the salivary microbiome in old age group

**Fig. 4:**
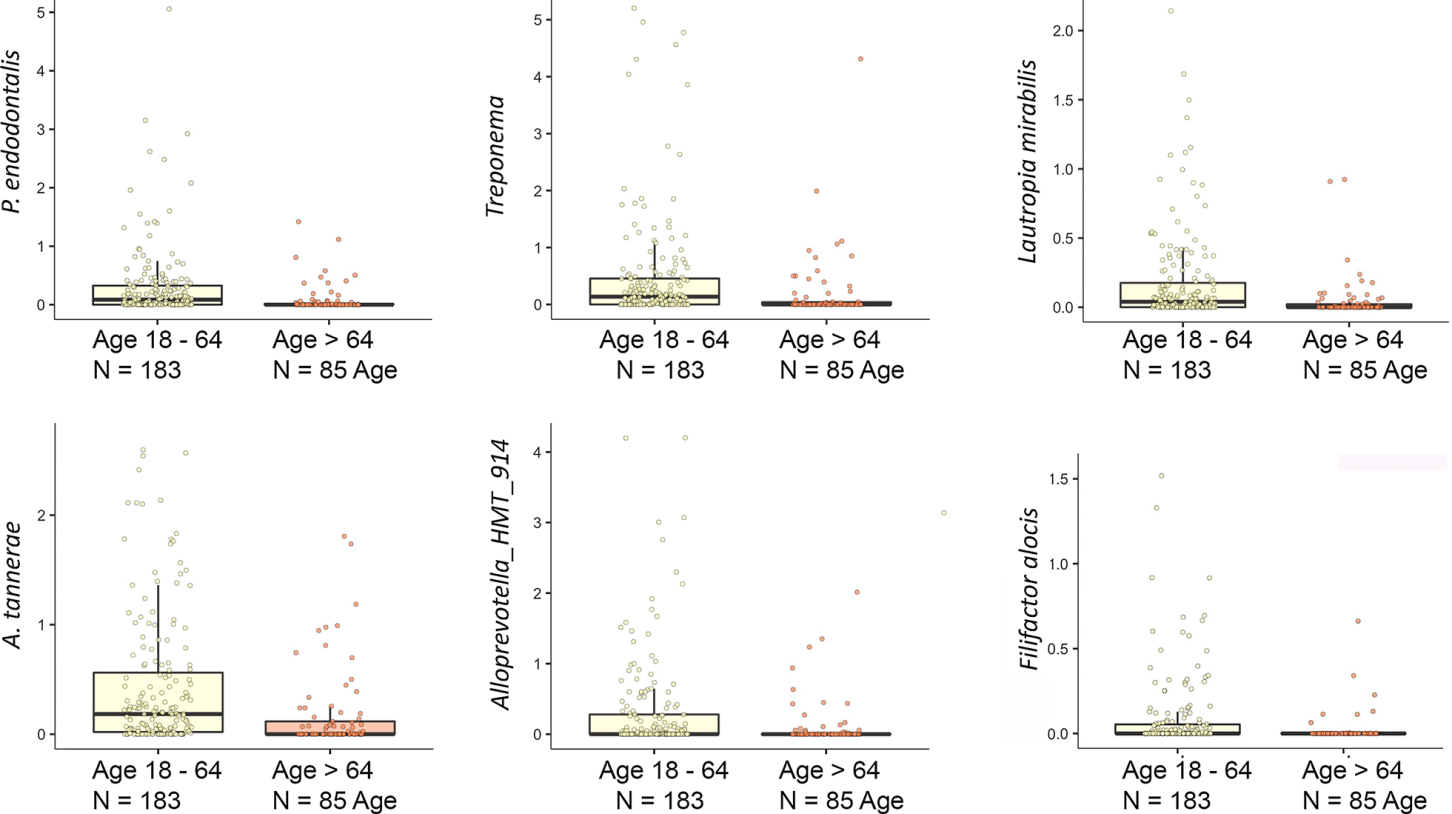
MaAsLIn analysis allowed the identification of taxa associated with old age versus middle and young age adults. Six taxa were shown to be at different levels in the old age group, FDR < 0.10, after adjusting for edentulism, tobaccos usage, periodontal disease, gender, caries, and number of medications used

Determination of multivariable associations was done using MaAsLin2 to identify differentially abundant taxa in the salivary microbiome given clinical covariates of age, caries, dentate status, gender, medication count, periodontal disease, and tobacco use. Most of the differentially abundant taxa were associated with edentulism in the comparison of the old age group versus all other subjects (Additional file 2: Supplemental Tables S1–S6). Furthermore, polypharmacy was associated with significantly altered abundance of 11 taxa, with a decrease in abundance of all but one taxa. After correcting for the clinical parameters previously listed, old age alone was associated with six differentially abundant taxa (Fig. 4). There was a lower relative abundance of *Porphyromonas endodontalis* and *Alloprevotella tannerae* (phylum: Bacteroidetes), *Filifactors alocis* (phylum: Firmicutes), *Treponema sp*. (phylum: Spirochaetes), *Lautropia Mirabilis* (phylum: Proteobacteria) and Ps*eudopropionibacterium sp._HMT_194* (phylum: Actinobacteria) in the old age group.

## Discussion

Edentulism, tobacco usage, and caries were significantly associated with variation of the beta diversity in the salivary microbiota (Fig. 3). This was expected as these conditions were already known to alter the salivary microbiome [19, 32]. More importantly, associations of age, periodontal disease, and gender also contributed to overall variations in the taxa. What is surprising is that none of the clinical parameters measured played a role > 1% as measured by the ADONIS analysis. Apparently, many factors each make small contributions to the diversity of the salivary microbiome. This conclusion is supported by an earlier study that focused on effects of diet and anthropometric measures, with no single factor contributing more than 2% to overall oral microbiome variation [33]. That this would also be true for oral disease, active caries, and periodontal disease may be because there are many niches in the oral cavity that are not affected by these diseases.

Old age contributes to differences in the oral microbiome reflected in the salivary microbiome. As Takeshida et al. observed earlier, the higher number of subjects with complete loss of teeth helped to decrease the alpha diversity in the old age group, likely by removing niches for many taxa [13] though differences in diet in this group may also contribute (Fig. 1). Similarly, the increase in periodontal disease seen in some old age groups would be thought to contribute to changes with age [2, 13]. Multivariable association analysis, as performed by MaAsLin2 allowed us to examine other contributors to oral microbiome changes [31]. Old age, age 65 and above, when adjusted for edentulism, current tobacco use, periodontal disease, caries level, medication count, and gender revealed 6 bacteria linked to this condition. These 6 taxa, at lower levels in the old age group, are found on or near tooth surfaces above or below the gum line. Three of the taxa, *Filofactor alocis*, *Porphyromonas endodontalis,* and *Treponema*, are known to be enriched with periodontal disease and one, *Lautropia mirabilis*, is enriched with periodontal health [34–37]. The two remaining taxa identified on the species level, *Alloprevotella tannerae* and Ps*eudopropionibacterium sp._HMT_194* are found on teeth but can also be found at mucosal sites [19, 38, 39]. Importantly, these differences were seen even after stratification of the data to only dentate subjects (data not shown). These differences were detected after correction for periodontal disease levels and caries in the different groups, suggesting an additional source. One might speculate that changes in inflammatory state or adaptive immunity in the oral cavity at large are responsible, given the changes in these systems that can occur with old age [2, 5, 40, 41]. When the results of this saliva study were compared to the recent comprehensive study of old age effects on subgingival microbiome of US woman [42] levels of two out of four taxa, measured in both studies, *Porphyromonas endontalis* and *Lautropia Mirabilis*, were lower in the > 70-year-old versus the 50–59-year-old group while they saw no difference in *Filifactor alocis* and *Alloprevotella tannerae* at that site. Other taxa identified as differentially abundant after correction in the old age group, shown in Fig. 1, have not been highlighted in saliva studies on aging in the past. This may be due to differences in diet, lifestyle, or environment in US versus those studies done chiefly in east Asia [11–13]. The exclusion of subjects with 1–20 teeth or dental implants in this study may also contribute to differences. Many niches in the oral cavity contribute to the saliva microbiome [43] and apparently many factors contribute to the identity of taxa in the salivary microbiome.

One may speculate on a number of changes that occur in the mouths of the post-65 group. This can include endogenous physiological changes of aging such as mucosal changes, changes in immune function, and changes in salivary flow. Because all 6 of the taxa that were differentially abundant in the old age subjects reside on or adjacent to hard surfaces, which are not known to change with age, one might speculate that microenvironmental changes in saliva, such as immune or inflammatory response to taxa on these surfaces may change with age [1]. It is difficult to compare the list of 6 species identified as different in the old age group, not explained by other clinical factors, to taxa known to be associated with xerostomia. Most of the studies on saliva microbiome and xerostomia focus on Sjogren’s Syndrome, an immune disease which is accompanied by xerostomia [44, 45]. Comparison to studies of Sjogren’s Syndrome and xerostomia using stimulated saliva for sample collection showed higher levels of *Treponema* and *Porphyromonas endodontalis* with these disorders, the opposite of what is seen in the old age group [45] suggesting that there is more to the difference in the old age group than low saliva flow (Fig. 3).

Another possible cause of old age differences, polypharmacy, or high level of medication use, is also a surrogate marker for chronic disease [46]. Others have suggested links between chronic disease and oral microbiome [47]. The ADONIS results suggest polypharmacy contributed minimally to the overall variation of the different taxa in the population (Fig. 3). However, MaAsLin2 analysis reveals several individual taxa that show differences in levels associated with high levels of medication use (Additional file 2). Thus, polypharmacy may contribute at a low level to individual taxa differences in the saliva microbiome in the old. Because differential presence of chronic disease in the aged may contribute to saliva microbiome differences a limitation of this study was that this was not recorded directly. Further weaknesses of this study are that there is no subject data on salivary flow which is expected to change with old age, nor on differences in diet not attributable to edentulism which may contribute to the salivary microbiome in old age. Future studies will include verified medical conditions in the analysis, volume of saliva flow, and diet makeup, to further discern the effect of aging on the oral microbiome.

## Conclusions

The measure of total salivary bacteria DNA concentration in the dentate suggest that overall levels per unit volume saliva were not lower in the old age group. Differences in specific oral microbiome taxa came with age, even after adjustment for increased levels of edentulism, polypharmacy, and additional possible confounders, periodontal disease, caries, tobacco use, and gender. These unexplained differences in specific salivary bacteria detected in this study, were all decreases in taxa associated with tooth surfaces, and occur with no obvious causes. Defining differences in oral bacteria with old age, the causes of these differences and possible effects on health by these bacteria, may aid in improving health in the elderly.

## Supplementary Information

**Additional file 1**. Supplemental Figure 1.

**Additional file 2**. Supplemental Tables S1-S6.

## Data Availability

The sequencing data from this study, is deposited in the National Center for Biotechnology Information Sequence Read Archive as PRJNA739492 and PRJNA674379.
