# Supplementary material for: Old age and other factors associated with salivary microbiome variation
Source: BMC Oral Health. 2021 Oct 4;21:490. doi: 10.1186/s12903-021-01828-1 (PMC8489047; doi:10.1186/s12903-021-01828-1)
Supplement: Supplementary file 1 — Additional file 1. Supplemental Figure 1. [file 12903_2021_1828_MOESM1_ESM.pdf]

Old age and other factors associated with salivary microbiome variation

Joel L. Schwartz, Natalia Pena, Nadia Kawar, Andrew Zhang, Nicholas Callahan, Steven J. Robles, Andrew Griebel, Guy R. Adami

Supplemental Figure 1 The Linear Discriminant Analysis Effect Size, LEfSe, was used to compare the abundance of bacterial species between the young/middle age group, age 18 -64 and the old age group, age 65- 94 (1). The cutoff for LDA was 3.0 and the FDR < 0.05. Even at this stringent cutoff, there are 28 taxa identified. There is no correction for potential confounders, tobacco usage, caries index, periodontal status, gender, medication usage, and edentulism. So the values may not be applicable to other populations that differ in these and other factors.

Reference

1. Segata N, Izard J, Waldron L, Gevers D, Miropolsky L, Garrett WS, et al. Metagenomic biomarker discovery and explanation. Genome biology. 2011;12:R60.

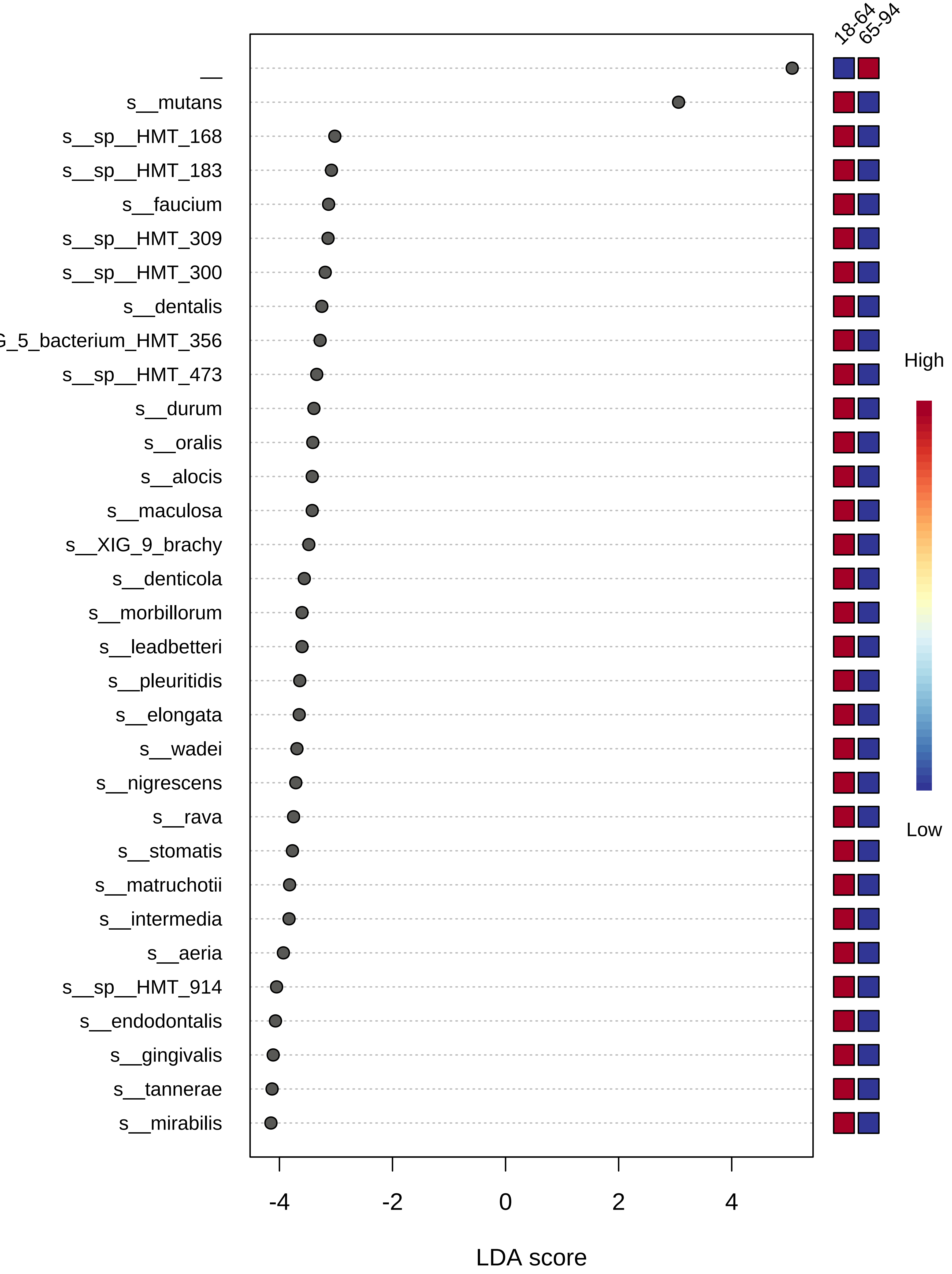

18-64
65-94
__
s__mutans
s__sp__HMT_168
s__sp__HMT_183
s__faucium
s__sp__HMT_309
s__sp__HMT_300
s__dentalis
G_5_bacterium_HMT_356
s__sp__HMT_473
s__durum
s__oralis
s__alocis
s__maculosa
s__XIG_9_brachy
s__denticola
s__morbillorum
s__leadbetteri
s__pleuritidis
s__elongata
s__wadei
s__nigrescens
s__rava
s__stomatis
s__matruchotii
s__intermedia
s__aeria
s__sp__HMT_914
s__endodontalis
s__gingivalis
s__tannerae
s__mirabilis
-4
-2
0
2
4
LDA score
High
Low
